# Streamlining Sensor Technology: Focusing on Data Fusion and Emotion Evaluation in the e-VITA Project

**DOI:** 10.3390/s25072217

**Published:** 2025-04-01

**Authors:** Michael McTear, Kristiina Jokinen, Sonja Dana Roelen, Muhammad Saif-Ur-Rehman, Mossaab Hariz, Jérôme Boudy, Christophe Lohr, Florian Szczepaniak, Rainer Wieching, Toshimi Ogawa

**Affiliations:** 1 School of Computing, Ulster University, Belfast BT155 1AP, UK; 2 Artificial Intelligence Research Center, National Institute of Advanced Industrial Science and Technology (AIRC/AIST), Tokyo 135-0064, Japan; kristiina.jokinen@aist.go.jp; 3 Institut für Experimentelle Psychophysiologie GmbH (IXP), 40215 Düsseldorf, Germany; s.roelen@ixp-duesseldorf.de (S.D.R.); m.saif-ur-rehman@ixp-duesseldorf.de (M.S.-U.-R.); 4 Institut Mines-Télécom (IMT), 91120 Palaiseau, France; mossaab.hariz@telecom-sudparis.eu (M.H.); jerome.boudy@telecom-sudparis.eu (J.B.); christophe.lohr@imt-atlantique.fr (C.L.); florian.szczepaniak@telecom-sudparis.eu (F.S.); 5 Business Information Systems and New Media, Universität Siegen (USI), Faculty III, 57068 Siegen, Germany; rainer.wieching@uni-siegen.de; 6 Institute for Development, Aging and Cancer, Tohoku University, Sendai 980-8577, Japan

**Keywords:** active and healthy ageing, sensors, emotion detection

## Abstract

This paper explores the use of sensor-based multimodal data fusion and emotion detection technologies in e-VITA, a three-year EU–Japan collaborative project that developed an AI-powered virtual coaching system to support independent living for older adults. The system integrates these technologies to enable individualized profiling and personalized recommendations across multiple domains, including nutrition, physical exercise, sleep, cognition, spirituality, and social health. Following a review of related work, we detail the implementation and evaluation of data fusion and emotion detection in e-VITA. The paper concludes with a summary of the key research findings and directions for future work.

## 1. Introduction

Over the past century, life expectancy has increased dramatically, leading to a rapid growth in the proportion of older adults in the global population. This demographic shift has been accompanied by a growing desire among seniors to maintain their independence and continue living in their own homes, creating an urgent need for innovative solutions to support active and healthy aging [1,2].

There is growing interest in the use of non-invasive interventions, such as those based on behavior change, as a means of disease prevention and health promotion. One example is mobile app-based health management services. Nevertheless, there is no comprehensive system that can address multiple domains at an individual level, especially among older people. A further challenge is the lack of professional and scientific information on the impact of app design and behavior change and well-being programs. This is a key issue that needs to be addressed when adopting digital technologies in the future.

This paper describes research into the use of sensor technologies and emotion detection in e-VITA, a three-year collaborative research and development project that was jointly funded by the European Union’s H2020 Programme (Grant Agreement no. 101016453) and the Japanese Ministry of Internal Affairs and Communication (MIC, Grant no. JPJ000595) [3].

The main aim of the e-VITA project was to promote active and healthy aging among older adults in Europe and Japan, supporting independent living and reducing the risk of social exclusion. To achieve this, a virtual coaching system was developed to provide individualized profiling and personalized recommendations across multiple domains including nutrition, physical exercise, sleep, cognition, and spirituality. The system identified risks in the user’s daily living environment by collecting data from external sources and non-intrusive sensors, offering support through natural conversational interactions with social robots.

In this paper, we focus on the application and evaluation of motion tracking and emotion recognition to enhance digital coaching and promote independent living for older adults. Other aspects of the virtual coaching system, including the Dialogue Manager responsible for natural conversational support, are discussed in [4,5].

The paper is structured as follows. Section 2 provides an overview of state-of-the art research on sensor-based multimodal data fusion and emotion detection technologies. Section 3 describes the implementation and integration of these technologies within the e-VITA project. Section 4 examines how sensor data and the Emotion Detection system facilitated proactive dialogues related to various aspects of daily living. Finally, Section 5 concludes with a summary of the key findings, discusses their implications, and offers suggestions for future research in AI-assisted healthy aging.

## 2. Related Work

Given the significant increase in life expectancy over the past century, it has become clear that traditional methods for the care of older adults have become unsustainable. This has led to an exploration of how new developments in Artificial Intelligence and related technologies can complement and enhance human-based approaches. In this section, we review related work in data fusion and emotion detection as applied in the e-VITA project.

### 2.1. Data Fusion

In general, a data fusion process can be described as a multidimensional approach aimed at enhancing the reliability of decision-making or event identification based on information from multiple sensors. In particular, it has been used in Human Activity Recognition (HAR), which is also its main purpose in e-VITA [6].

**Figure 1:**
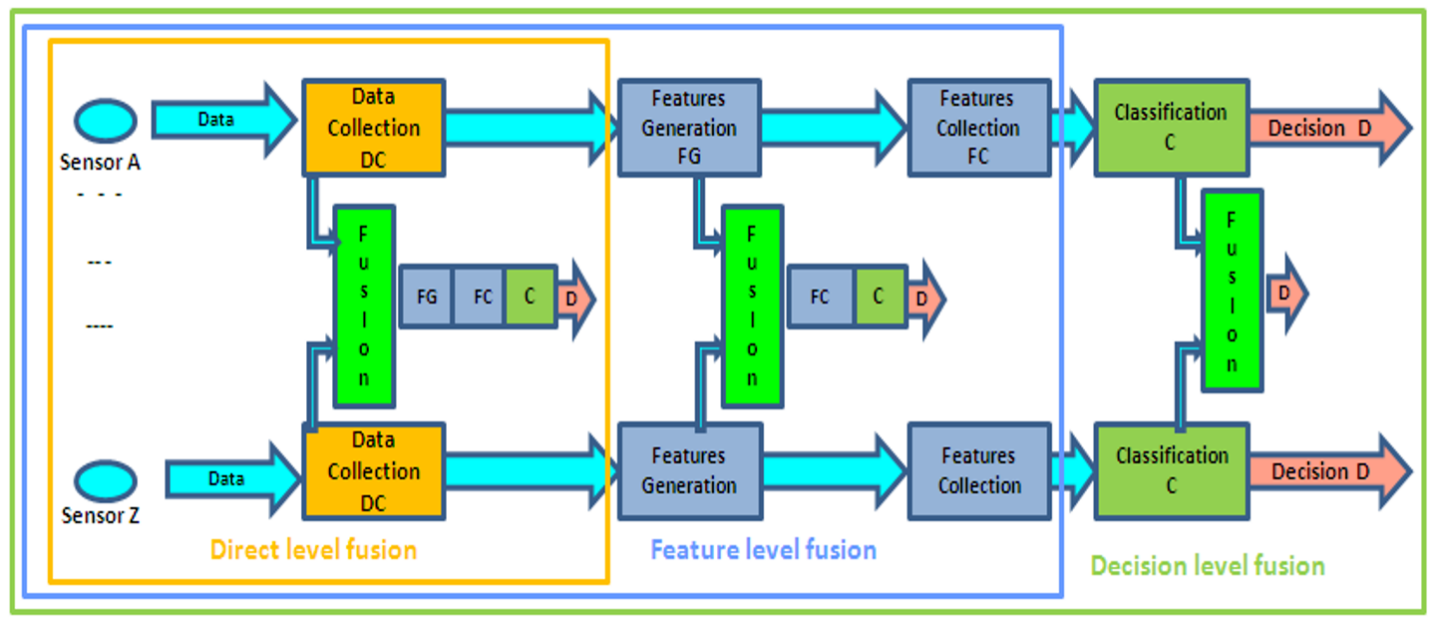
Different levels of data fusion. Source: [10].

The data fusion process can be implemented at different levels, depending on the level of information used in the fusion process, as illustrated in Figure 1.

The Data or Observation Signal Level involves information from various environmental sensors. Observations are typically organized as vectors, which can represent either preprocessed signals (direct-level fusion) or analyzed signals (feature-level fusion). Furthermore, raw digitized signals can be used as, e.g., in a series of binary impulses from a passive infrared (PIR) detector. The vectors are then processed using identification or classification methods such as Kalman filtering, Neural Networks, or Support Vector Machines, and the methods are applied globally to the dataset to determine the probability of normal or abnormal events occurring.

The Decision System Output Level involves the outputs of different decision systems operating in parallel on complementary or redundant signals, such as classifiers or expert systems. Results are usually expressed as recognition scores (likelihood or a posteriori probabilities). At this level, score-fusion techniques are applied. These techniques were originally developed for image and speech processing, for example, in the fusion of phonetic signals and viseme-based pattern recognition, where visemes represent visual speech elements such as lip movements [7,8].

The fusion process can interpret signal or recognition score data in several ways, including competitive, complementary, or redundant approaches, depending on the fusion architecture and the correlation properties of the different sources or modalities involved [9]. Typically, the data or decision outputs being fused are homogeneous, especially in competitive data fusion scenarios.

However, multimodal data may not be homogeneous at the same fusion level. For instance, data might come from diverse sources, such as pattern-matching approaches (e.g., HMM, GMM) and rule-based processing, or involve different structures (e.g., continuous versus binary signals). Therefore, it is beneficial to consider fusion approaches that can integrate different data types without necessarily considering their structure or level of abstraction.

To address heterogeneity in signal and data processing, various approaches have been developed, such as Fuzzy Logic combined with rule-based systems, Bayesian networks, and Evidential Networks based on Belief Theory [9,11,12]. For example, Bayesian score fusion, which involves multiplying all class posterior probabilities from parallel statistical pattern recognition processes, was applied to bi-modal audio-visual recognition by [7,8].

The experiments highlighted the need for heterogeneous data fusion due to the diverse nature of the data sources, including the following: actimetry data (movement signals), sound signals, and vital parameters (heart rate). To address this, heterogeneous unsupervised data fusion approaches such as Fuzzy Logic, introduced by Zadeh in 1965 [13], and Evidential Networks based on the Dempster–Shafer theory by Shafer in 1976 [14], have been employed. These methods not only handle data heterogeneity but also mitigate issues related to the scarcity of training data for supervised classification, particularly for rare events like falls or distress, and to a lesser extent, daily activities [15].

**Figure 2:**
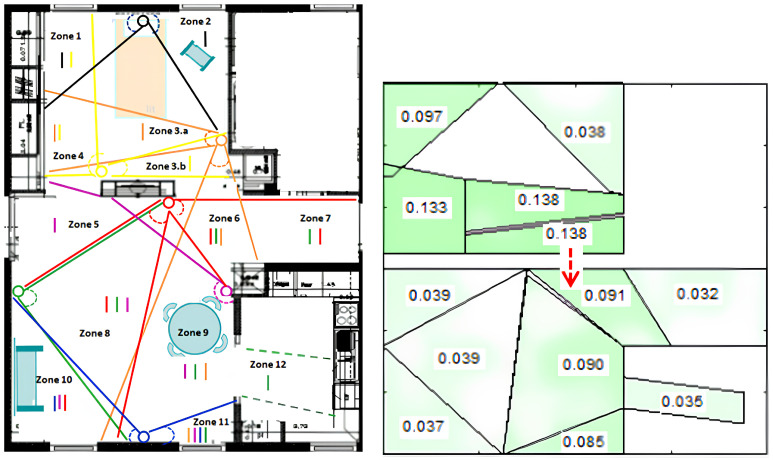
PIR sensors-based localization using the Dempster–Shafer theory [16].

In a recent research project on ADL (CoCaps FUI-project), the authors in [16] developed a localization system using PIR sensors and the transferable belief model based on the Dempster–Shafer theory. This system provides accurate localization within the user’s home environment and correlates it with the user’s ADL (see Figure 2).

Over the past decade, deep machine learning approaches have revolutionized pattern recognition and fusion techniques, offering significantly improved performance compared to traditional methods. These advancements primarily involve specialized neural network architectures, such as Convolutional Neural Networks (CNNs), Recurrent Neural Networks (RNNs), and Long Short-Term Memory Networks (LSTMs). Recently, Deep Neural Networks have been applied to actimetry signal acquisition, particularly in wearable tracking systems for fall prevention and Activities of Daily Living (ADL) monitoring, as demonstrated in the works of [15,17,18,19]. Additionally, Ref. in a comprehensive state-of-the-art review LSTM approaches are compared with traditional Kalman and Extended Kalman Filtering (EKF) methods, highlighting the limitations of the latter due to their reliance on noise assumptions [18].

In the context of the e-VITA project, these advanced approaches have been explored and applied to data fusion problems involving multiple data inputs. For example, localization signals from a PIR sensor network and actimetry signals from wearable devices (including 3D accelerometery and inertial sensors) have been integrated for interaction with Knowledge Graphs and Dialogue Manager systems. Moreover, a definition of data fusion was introduced within the e-VITA context in which two paradigms were proposed based on combined algorithmic and architectural principles ref. [4]. This approach involves multiple algorithmic principles interfacing with a kernel that manages various data sources. Consequently, the data fusion platform (see below Section 3.2) is designed to implement this architectural principle, effectively embodying the conceptual framework of data fusion.

### 2.2. Emotion Detection

The detection of emotion in conversation is a crucial aspect of human–computer interaction and affective computing. Accurately identifying emotions during conversations can enhance the quality of interactions, improve user experiences, and provide valuable insights into human behavior and mental states.

Computers and robots therefore need to be taught how to identify, understand, and express emotions to interact authentically with users [20]. A coach capable of detecting and reacting to the user’s emotional state will increase the user’s general acceptance as well as the success of the coach’s interventions [21,22]. Recent studies examine the integration of emotion detection in virtual assistants and chatbots to enhance user experiences, employing various modalities such as natural language approaches, video, and speech, to recognize basic emotions like anger, joy, and sadness [23,24]. The mental health chatbot SERMO was designed to help users regulate their emotions through cognitive behavioral therapy (CBT) techniques [23]. The chatbot functions as a mobile application that integrates a chatbot prompting users to report their daily events and emotions. The chatbot automatically detects the user’s basic emotions based on a natural language and lexicon-based approach and suggests personalized activities or mindfulness exercises accordingly. The application also features an emotion diary, a list of pleasant activities, mindfulness exercises, and information about emotions and CBT. The usability of SERMO was evaluated, with positive feedback regarding its efficiency, perspicuity, and attractiveness.

Applications like the personal assistant “Edith” leverage deep learning techniques to detect emotions and support users experiencing social anxiety or depression [24]. This assistant adjusts its responses based on the detected emotional state, providing more empathetic and supportive interactions. Such dynamic interaction not only enhances user experience but also addresses emotional needs, rendering the assistant more effective and personable. This research underscored challenges in achieving high accuracy in emotion recognition due to subtle differences in facial expressions and potential dataset biases. Additionally, maintaining user privacy and data security is identified as a critical challenge. The specific challenges of audio and video emotion recognition in older adults were addressed in a study that investigated how virtual agents can accurately detect and respond to emotional expressions in this demographic [25]. The study highlighted the variability in facial expressions and the necessity for tailored interaction strategies. It also pointed out the difficulty of recognizing emotions in older adults due to limited relevant datasets, calling for more comprehensive evaluations to ensure system reliability.

In an earlier study on adaptive dialogue interventions, an embodied conversational agent was developed for mindfulness training and coaching through natural language dialogues [26]. The “Virtual Mindfulness Coach” could initiate or change topics, focusing on “affect-adaptive interaction”, where the coach’s responses were tailored based on the student’s emotional state. A pilot evaluation comparing the effectiveness of this virtual coach-based training with a self-administered training program using written and audio materials showed that the virtual coach-based training was more effective.

## 3. Sensor and Emotion Detection Technologies Used in the e-VITA Project

A key component of the e-VITA system is its ability to enhance user interaction through a virtual coaching framework. It incorporates real-time data fusion from multiple sources—such as smart home sensors and wearable devices—to generate personalized insights and proactive notifications. Additionally, the system employs advanced emotion detection models to assess user emotional states based on speech analysis, enabling more empathetic and adaptive interactions.

This section explores the core functionalities of the e-VITA system, detailing its data fusion platform, proactive notification system, and emotion detection capabilities. Through these innovations, e-VITA aims to provide a comprehensive and intelligent support system for users, particularly in promoting well-being and safety.

### 3.1. The Digital Enabler Platform

**Figure 3:**
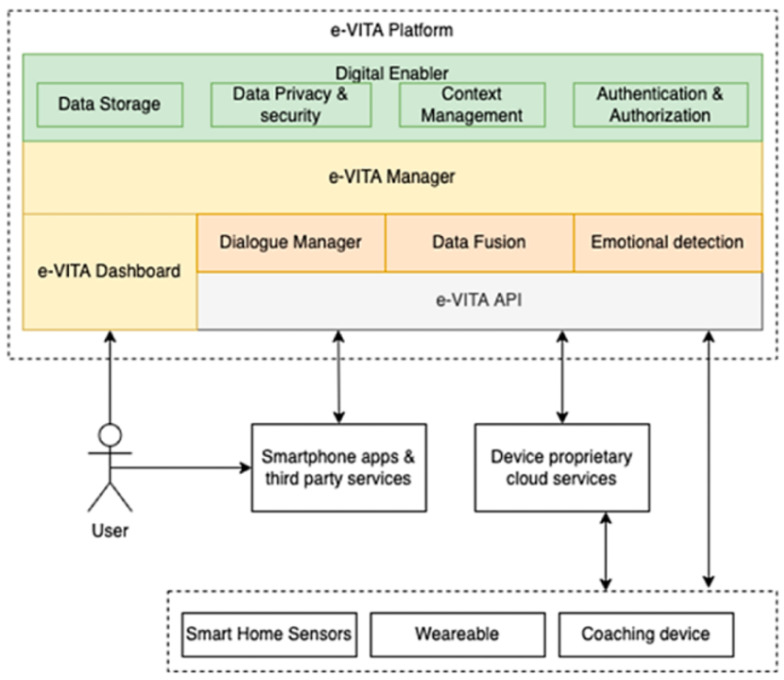
High-level view of the e-VITA platform. Source: [4].

The e-VITA system is based on the Digital Enabler platform [27]. The platform is designed to handle data from heterogeneous devices, including smart home sensors such as temperature, humidity, and intrusion sensors, and wearable devices such as smart watches, smart bands, and smart rings. It also enables interaction with coaching devices such as social robots and holograms. Additionally, the platform provides capabilities for data storage, context management and overall communication with external systems. Special attention is paid to data privacy and security, as well as authorization. Figure 3 shows a high-level view of the e-VITA platform.

The e-VITA platform enables the integration between the devices and the primary components of the e-VITA virtual coach—the Dialogue Manager, the Multimodal Data Fusion module, and the Emotion Detection module. The user is registered as the user of the system and a particular interface device through the dashboard, through which the user can also provide personal properties and features which affect the interaction. We will not go into the details of the platform as this is beyond the scope of the current paper. For a detailed description, see [4].

The platform operates as a messaging system—the system maintains message queues and messages are sent to the appropriate components while the information is processed within the individual components like the Dialogue Manager (DM) and the Emotion Detection System (EDS), according to their own specifications. More specifically, the user interacts with the system by typing in the text input on the interface device or speaking into microphone of the selected social robot (in the latter case, the spoken utterance is transformed into text using the Google Automatic Speech Recognizer). The text input is passed further to the Digital Enabler where it is augmented, if applicable, with labels representing additional information fused from sensors and the emotion detection system. The response is generated by the Dialogue Manager as a reaction to the user input, and it is sent back to the Digital Enabler, which processes the response and sends it to the selected interface device (in case of the robot, the robot’s Text-to-Speech system is used to output the system response as speech). In addition to the interactions initiated by users, system-initiated dialogues can be triggered through the notification management system, for example, to prompt the user about an upcoming appointment or to remind about taking exercise, see Section 3.2. The Dialogue Manager is described in further detail in [5,28].

### 3.2. Data Fusion Platform

The main objective of the Data Fusion Platform (DFP) is to provide higher quality information according to the different multimodal data sources, and as mentioned, in the e-VITA project, the specific objective is to provide human activity recognition (HAR). Our data sources are as follows: inertial information (accelerometer, gyroscope, and magnetometer) from smartphone; location (latitude, longitude, altitude, and speed) from smartphone; motion, or intrusion detection with DeltaDore (EU)/EnOcean (JP) sensors; indoor climate with Netatmo Smart Indoor Air Quality Monitor, measuring temperature, humidity, CO_2_ and noise level; the EnOcean ETB-RHT, measuring temperature and humidity; and health information (such as bpm, sleep, and steps) with the Huawei smart band.

In order to build initial models from smartphone inertial or motion/intrusion sensors, we used the following two public datasets:

- Ambient sensors: “Human Activity Recognition from Continuous Ambient Sensor Data” from the University of California, Irvine (UCI);
- Smartphone: “KU-HAR: An Open Dataset for Human Activity Recognition” from Medeley data.

Based on the data collected from the different sensors, the DFP calculates for each user a set of real-time labels and stores them in the Digital Enabler (DE), as follows:

- Gait analysis: walk, run, lie, sit, stand;
- Human activity analysis: cooking, resting activity, enter home, leave home, *…*;
- Games: number of steps based on the mobile application;
- Time spent in each location of the user’s home;
- WBGT value calculated based on temperature and humidity.

These labels are used to trigger proactive dialogues based on the situation of the user.

#### 3.2.1. Heat Stroke Warnings

A key safety consideration addressed by environmental monitoring is the risk of heat stroke. Heat stroke, characterized by a sudden increase in body temperature due to exposure to excessive heat or a combination of heat and humidity, poses a significant threat, particularly to older people, as it overwhelms the body’s natural heat-regulation mechanisms. The technical approach adopted to mitigate this risk involves using the Wet-Bulb Globe Temperature (WBGT) as a crucial indicator for assessing the degree of heat stress. WBGT serves as the basis for developing preventive guidelines. More specifically, for indoor environments, the WBGT index is computed using the following formula:

WBGT=0.567·T+0.393·H+3.94

where T is the temperature (°C), and H is the relative humidity (%). This formula allows the DFP to tailor suggestions based on real-time sensor data, offering timely alerts for potential heat stroke risks [29].

#### 3.2.2. Triggering Proactive Conversations

**Figure 4:**
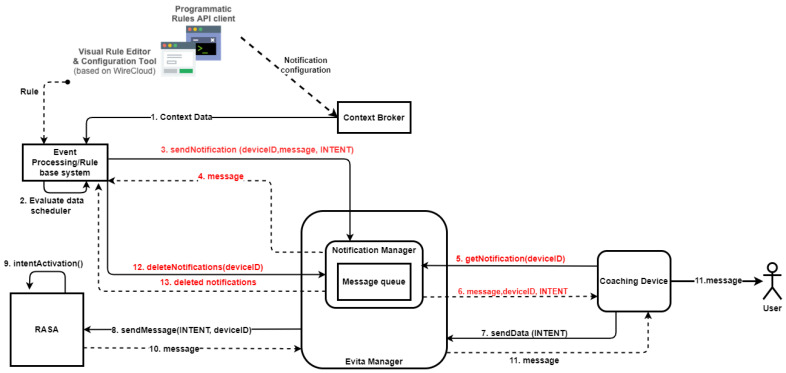
Rule-based decision for triggering specific dialogue scenario.

To initiate proactive dialogues based on the predicted user situations, the e-VITA system employs an Event Processing/Rule-based approach. This is implemented as a notification system which is integrated into the communication scheme among the different components of the e-VITA system. The architecture is presented in Figure 4, and the components in the notification system include the following:

- Event Processing (EP)/Rule-based component: Identifies specific data patterns using sensor data or events based on rules to trigger notifications or provide textual advice to the user;
- RASA Dialogue Manager: Responsible for activating dialogues based on specific intents;
- Notification Manager (NM): A specific component of the DE, more specifically, a part of the e-VITA Manager component, managing the notification flow and message queues;
- Coaching Device (CD): The device used by the user to engage in dialogue with e-VITA after a specific event occurs.

The general flow of the notification system is as follows:

1. The EP identifies a specific event related to a data pattern (e.g., high temperature) or time schedule;
2. The EP evaluates the data and identifies if any specific rule is met;
3. If the conditions are satisfied, the EP sends a notification request (“sendNotification(deviceID, message, INTENT)”) to the Notification Manager (NM);
4. The NM places the message in the message queue and send back a conformation about the creation of the notification;
5. The CD periodically queries the NM to retrieve relevant notifications using the “get-Notification(deviceID)” method;
6. The NM receives the request, checks the queue, and responds with the corresponding “message”, “deviceID”, and “INTENT”;
7. The CD sends the “INTENT” to RASA via the e-VITA Manager (“sendData(INTENT)”). The CD does not display any message to the user at this stage;
8. The message is sent to RASA as the usual flow with the “deviceID” of CD;
9. RASA activates the intent;
10. RASA responds with a message, continuing the dialogue flow;
11. The message is delivered to the user via the CD.

The e-VITA system employs Perseo [30] as its brain for processing events. Perseo listens to changes, follows rules, and takes actions. Conceptually, it serves as a smart organizer that understands events in a simple language and reacts accordingly by triggering actions and dialogues. Perseo rules follow a simple JSON structure made up of the following three mandatory key-value fields: ***name***, ***text***, and ***action***. The structure of these rules is sketched in the following JSON code:



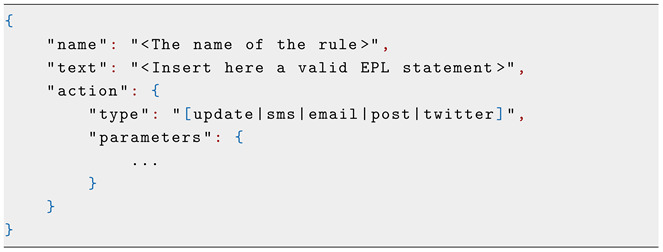



The ***name*** field refers to the name of the rule, and it is used as rule identifier. It must start with a letter, and it can contain digits (0–9), underscores (_), and dashes (-). Their maximum length is set to 50 characters.

The ***action*** field states the action to be performed by Perseo when the rule triggers. We can also use an array of action objects if needed. Each of those actions will be executed when the rule is fired.

The ***text*** field contains the valid EPL statement to be sent to the Esper-based core rule engine. The value of this field must follow the EPL syntax.

A sample rule in e-VITA has the following structure:



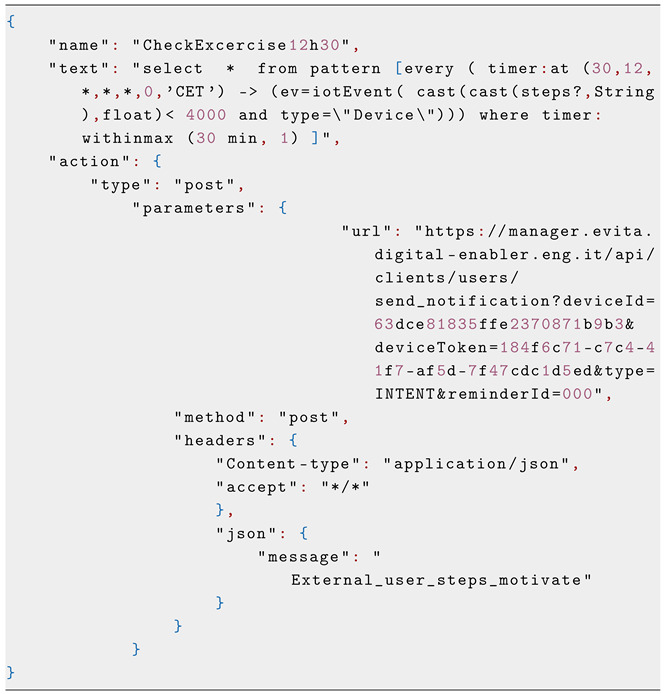



The **name** is an identifier for the rule, enabling it to be selected and configured according to the user’s preferences.

In e-VITA rules, all **actions** are HTML POST requests to the NM with the identifier of the CD (deviceId,deviceToken) for which the notification is intended. The *message* in the POST query contain the *INTENT* (in our case, **External_user_steps_motivate**) that the CD should send to RASA to start the correspondent dialogue.

The EPL clauses in e-VITA can be time-based, sensor event-based, or both. The following **text** field is extracted from the above e-VITA rule:

$$\begin{array}{cc} \mathrm{select} & *,\mathrm{from}\;\mathrm{pattern}[\\ \; & \mathrm{every}(\mathrm{timer}:\mathrm{at}\left(30,12,*,*,*,0{,}^{\prime }CET^{\prime }\right)\\ \; & \to (ev=iotEvent(\mathrm{cast}\left(\mathrm{cast}\left(steps?,\mathrm{String}\right),\mathrm{float}\right)\\ \; & <4000\mathrm{and}type=’’Device’’)))\\ \; & \mathrm{where}\mathrm{timer}:\mathrm{withinmax}\left(30\;\min,1\right)] \end{array}$$

This EPL clause monitors the value of the steps made by the user by 12:30 and will trigger a dialogue to motivate the elder to take more exercise if it is below 4000. The threshold and the time can be personalized according to the user’s preference.

The DFP in the e-VITA project offers better flexibility than traditional data fusion methods, as described in Section 2.1. Unlike conventional approaches that focus on homogeneous data, the DFP integrates heterogeneous multimodal sources, including inertial sensors, environmental monitors, and health-tracking devices. By leveraging real-time labels, it enhances HAR and enables proactive, context-aware interactions.

This flexible, heterogeneous fusion approach enables a more comprehensive understanding of user behavior, overcoming data diversity and real-time variability challenges. The DFP evolves beyond conventional fusion paradigms, offering a robust, real-time decision-making system that improves personalized support in home healthcare and well-being applications.

### 3.3. Emotion Detection

Automatic emotion detection facilitates the development of emotionally aware and empathetic technology. As part of its affective computing capabilities, the e-VITA platform incorporates an Emotion Detection System (EDS) to detect emotions from the user during the interaction with the coaching system based on speech.

Typically, audio-based emotion recognition predominantly focuses on speech in terms of paralinguistic features [31]. The following four broad types of speech data variables can be analyzed according to [32]: continuous acoustic variables (pitch/F0 height and range, duration, intensity, and spectral makeup), pitch contours (pitch variation in terms of geometric patterns), tone types (intonational phrases or tone groups), and voice quality (auditory descriptions such as tense, harsh, and breathy). In previous research, different types and shapes of various parts of a tone were found to be relatable to different emotions [32].

A use case was developed specifically for this speech interaction, facilitating natural and flexible engagement. In this scenario, the coaching system greets the user upon entry, asks the user about their day, and utilizes the Speech Emotion Recognition (SER) model implemented in the EDS to assess the user’s emotional state based on their verbal response. For this reason, a dialogue function entitled “External_how_was_your_day” was designed specifically for speech-based interaction, see Section 4.2.

The EDS was extended in the second phase of the project by integrating video-based emotion recognition (VER) models to enable the analysis of facial expressions in video data. In the field of video-based emotion recognition, analyses of facial expressions are most common. Here, emotion is often expressed by subtle changes in one or a small set of discrete facial features. For example, anger might be displayed by a tightening of the lips, or sadness by lowering the corners of the mouth [33]. In general, facial expressions are based on changes in various muscular action units, coded in the Facial Action Coding System (FACS) developed by [34]. They defined 44 action units, of which 30 are anatomically related to the contractions of specific facial muscles as follows: 12 are related to the upper face and 18 to the lower face. The action units can be analyzed individually as well as in combination [35].

The VER model is meant to be seen as a complementary tool for providing a more comprehensive assessment of the user’s emotional state. While VER adds valuable capability, its deployment is best suited for scenarios where users can be positioned directly in front of the system.

#### 3.3.1. Emotion Detection Model for Speech Signals

The EDS is a deep neural network model for speech emotion classification, constructed from a two-dimensional time distributed convolutional neural network (2D-CNN) and a Long Short-Term Memory (LSTM) network. The model processes log-mel spectrograms of audio signals, using four local feature learning blocks (LFLBs) to extract temporal and spectral features, followed by a LSTM layer to model temporal dependencies. This architecture allows the system to learn both local and global features from the input data.

#### 3.3.2. Emotion Detection Models for Facial Expressions

For VER, we developed and tested the following two frameworks: the Conventional Framework and Shallow Convolutional Neural Networks.

##### Conventional Framework

This framework uses the Facial Action Coding System (FACS) [36] in conjunction with a Support Vector Machine (SVM) [37]. FACS are mapped to action units (AUs) by using the open-face behavior analysis toolkit [38]. The extracted AUs are treated as features which are further used to train the SVM.

##### Shallow Convolutional Neural Network

This approach uses Convolutional Neural Networks (CNNs) [39] which are state-of-the-art deep learning models for image recognition tasks [40]. CNNs learn and automatically extract the generalized features from the given images using learned kernels; hence, manual curation for feature extraction is not required. In the e-VITA project, we used CNNs for meaningful feature space learning, and a dense layer for the mapping of feature space to decision space. The end-to-end Shallow CNN has four convolutional and pooling layers for feature extraction and two fully connected (dense) layers for mapping the features from latent space to decision space.

Preprocessing of the input image is done in two stages, as follows: In the first stage, extraction and face cropping are performed with the Python open source MediaPipe library [41]. In the second stage, the cropped faces are transformed from RGB to grey scale, and the images are resized to 48 × 48 × 1.

**Figure 5:**
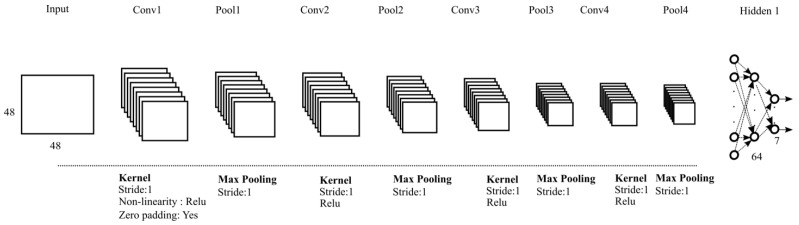
Architecture of a Shallow CNN. It has four convolutional layers and pooling layers with two fully connected layers.

The Shallow CNN architecture consists of four convolutional and max-pooling layers (see Figure 5). First the cropped and resized faces (48 × 48 × 1) are fed to the first convolutional layer, which has 32 kernels (3 × 3), followed by a batch normalization layer [42]. In the second convolutional layer, the resultant features are then further convolved with 32 kernels, followed by another batch normalization layer and the down-sampling of the features with a max pooling (2 × 2) layer. To further minimize the impact of overfitting, drop-out regularization (0.5) [43] is employed. In the third convolutional layer, features from the second layer are convolved using 64 kernels (3 × 3) followed by batch normalization. The fourth and final convolutional layer contains 64 kernels (3 × 3), batch normalization, max pooling (2 × 2), and dropout (0.5).

Convolved features from the fourth layer are fed into the first fully connected (dense) layer with 64 neurons with batch normalization and dropout (0.5). The second dense layer is the classification layer (softmax), which contains seven neurons, representing the emotions of interest. For all the convolutional and the first dense layers, non-linearity is introduced with rectified activation linear unit (Relu) [44].

### 3.4. Datasets

#### 3.4.1. Training and Validation Datasets for VER Models

The Training Dataset for the VER model uses the publicly available Kaggle Facial Expression Recognition (FER-2013) dataset [45] for training and validation. FER-2013 contains 48 × 48-pixel grayscale images of cropped faces. The training set of the FER-2013 dataset consists of 28,709 examples, whereas test set of FER-2013 consists of 3589 images of the seven following emotions: Angry, Disgust, Fear, Happy, Sad, Surprised, and Neutral.

The Validation Dataset of the model consists of 3589 test images of the FER-2013 dataset. The validation accuracy of the employed Shallow CNNs model on the FER-2013 test dataset is 61%, the fine-tuned Mobile net V3 is 50%, and the conventional framework is 43%. The fine-tuned Mobile net V3 achieved up to 88% training accuracy and 50% validation accuracy, which is a clear indication of overfitting. Therefore, we did not include the pre-trained model for further analysis.

#### 3.4.2. Evaluation Datasets for VER Models

The Trained VER models were evaluated on the two different Datasets of senior people: the LivingLab Datasets, and the Corpus of Interaction between Seniors and an Empathic Virtual Coach in Spanish, French and Norwegian (CISEVCSFN) [46].

The LivingLab Dataset is a video dataset of senior people created in the LivingLab studies of the University of Siegen. The first testing of the VER-model used the e-VITA age-specific subsample of this data. Data Collection was done during a living lab session conducted at the University of Siegen, where a video data corpus comprising eight participants was generated. During the session, the participants engaged in a thirty-minute interaction with an android robot. The initial phase of interaction was carefully crafted as a Wizard-of-Oz setup [47], wherein the android’s utterances were under the control and authorship of the researchers. The participants were recorded with an external camera on a tripod standing next to the Android Robot. All the videos were post-processed and re-encoded to .mp4 format with around 30 frames per second and maintaining the original aspect ratio.

The videos were labeled manually by one expert annotator. For the video emotion annotations, the whole video was considered, i.e., video segments that correspond to the user speaking, and video segments that correspond to the user listening to the virtual coach. The videos were annotated with temporal start-end marks for each emotion using ELAN software (ELAN Version 6.2). Only surprise, anger, happiness, and neutral have been annotated, as other emotions did not occur within the interaction between the seniors and the Android Robot.

**Figure 6:**
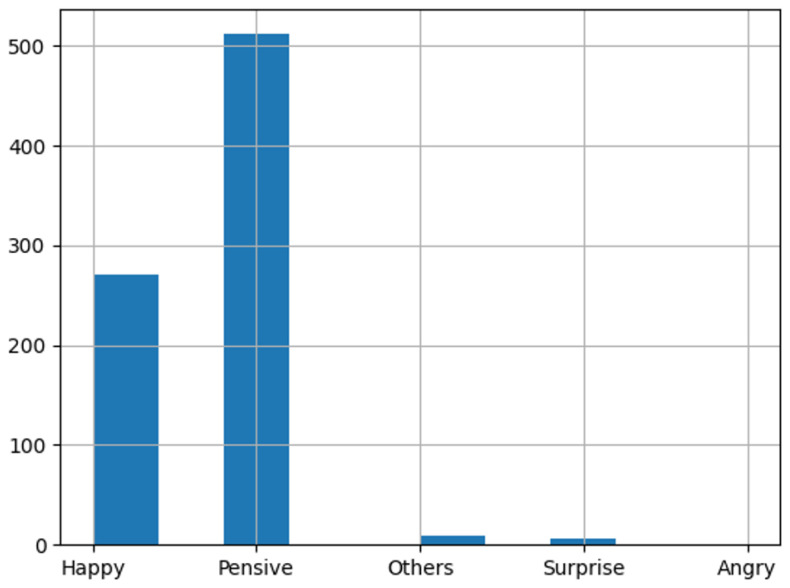
Distribution of the test data on agreed snippets of both the annotators.

The CISEVCSFN dataset consists of the following emotion classes: Angry, Happy, Sad, Pensive, Surprise, and Others. It was labeled by two annotators, who sliced the full videos of each participant into small chunks and assigned one of the above-mentioned labels to each chunk. It was possible for both the annotators to agree and assign the same label to one chunk, or both the annotators could disagree and assign different labels to the chunk, or one of the annotators could not distinguish the change of emotion and miss the chunk and could not assign the label to the given chunk. For our analysis, we only considered the video chunks where both the annotators agreed and assigned the same label. The distribution of the clean data is given in Figure 6. Concretely, both annotators assigned 824 snippets (Pensive: 531; Happy: 274; Others: 11; Surprise: 7; Angry:1; Sad: 0) with the same emotion label.

### 3.5. Evaluation Procedure

We used the AU in combination with the SVM-based model (conventional method) and the Shallow CNN on the LivingLab data corpus. During annotation, a time interval (with starting and ending time) of a video was labeled with one of the above defined seven basic emotions. Both the models were trained on static images, and to generate one prediction during the annotated time interval, frames were extracted after every 33 msec and a simple criterion was introduced to assign a label to the given video by calculating the mode of predicted outputs of all the frames of a video. Mathematically, this operation is represented in the equation below.

$$y_{pred}\left(video\right)\;=mode\left(y_{pred_{frame}}\right)$$

Next, the mode operation was applied to all the predictions of the extracted frames to get one final prediction. We evaluated the trained model on each recording session individually. The evaluation performance of both models on five selected recording sessions is shown in Table 1. Shallow CNN outperforms its counterpart (AU+SVM) on four out of five recording sessions. Overall, the Shallow CNN achieves 48.95% mean accuracy and AU+SVM achieves 21.92% accuracy.

In addition, we evaluated the trained Shallow CNN on CISEVCSFN, which contains a different set of example emotions. Originally, the Shallow CNN is an image classification model. Here, however, we have videos of small duration to classify. Therefore, the criteria defined above were used to extract the single label from the given video. Otherwise, slight adjustments in the name conventions were made, where we renamed “Neutral” as the “Pensive” class, and “Disgust” and “Fear” were combined and renamed “Others”.

### 3.6. Emotion Evaluation Results

#### 3.6.1. VER Evaluation

**Figure 7:**
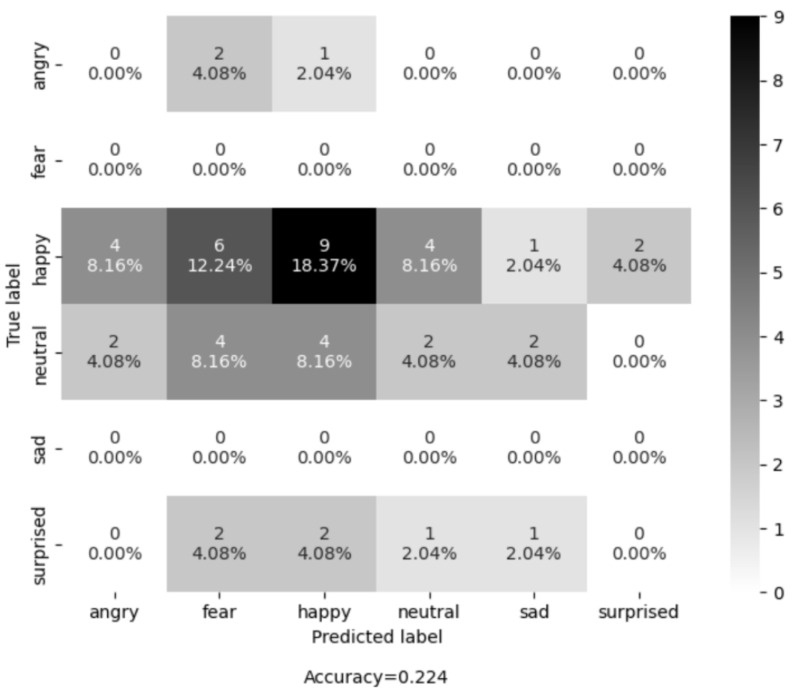
Confusion matrix of the AU-based model on the LivingLab senior people dataset.

**Figure 8:**
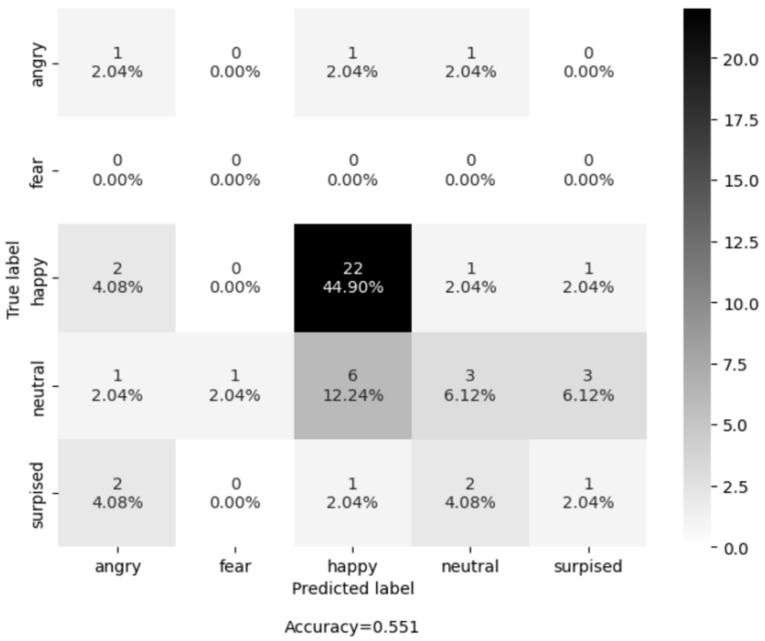
Confusion matrix of the CNN-based model on the LivingLab senior people dataset.

**Figure 9:**
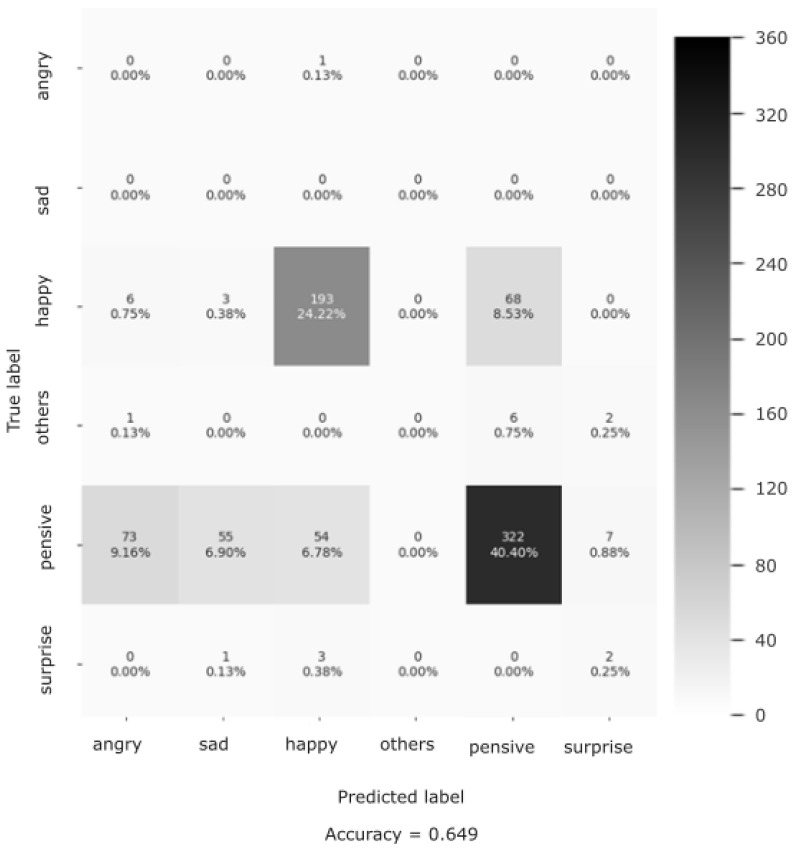
Classification performance (confusion matrix) of the Shallow CNN on the CISEVCSFN.

Additionally, to report the results in a more transparent way, we used confusion matrices (see Figure 7 and Figure 8) on all the examples of all the recording sessions. Most examples of the evaluation dataset belong to the class “Happy”. The Shallow CNN correctly classified 22 examples, while AUs + SVM only correctly classified 9 examples out of a total of 26 examples. The class with the second highest number of examples is “Neutral ”, where the Shallow CNN correctly classified 3 and AU + SVM correctly classified 2 out of the 14 examples. The results shown in Table 2 and Figure 9 suggest that Shallow CNN is a better option than its counterpart, with an evaluation accuracy of 55.1% versus an evaluation accuracy of 22.4% demonstrated by the AUs + SVM model. However, it also suggests that the Shallow CNN is biased towards the “Happy” class.

In conclusion, the Shallow CNN model shows promising results since it outperforms the AU+ SVM model. However, the current version of Shallow CNN has certain limitations. Firstly, it exhibits a bias towards the “Happy” class, leading to the misclassification of neutral faces as happy faces. Secondly, its evaluation was conducted on a small subset of the available evaluation dataset. Thirdly, the evaluation dataset was labeled by only one expert annotator. To address these limitations and pave the way for future improvements, two potential approaches are recommended. Firstly, fine-tuning the Shallow CNN on both the University of Siegen training dataset and other datasets can enhance its performance. Secondly, annotating the data using the expertise of at least two experts would ensure more reliable and robust evaluations.

#### 3.6.2. CISEVCSFN Evaluation Results

The evaluation performance of the Shallow CNN on the CISEVCSFN is shown in the confusion matrix (Figure 9). Here, the confusion matrix has the following classes: Angry, Sad, Happy, Others, Pensive, and Surprise, Pensive and other emotions were not defined in the training dataset, so we renamed them as “Neutral” and merged the “Disgust” and “Fear” classes and called them “Others”.

The overall classification accuracy of the model is 64.9%. In addition to classification accuracy, we reported the precision (78%), recall (65%), and f-1 scores (71%) as additional evaluation metrics (see Table 2). These results demonstrate the high generalization quality of the Shallow CNN on the data of the people in the older-aged group.

## 4. Sensor Models in the Coaching Dialogues

### 4.1. Data-Driven Dialogues for Proactive Interactions

As stated earlier, the e-VITA virtual coach delivers accurate information on various aspects of daily living, offering proactive advice based on environmental sensor data. These proactive dialogues covered several domains such as daily activity, nutrition, safety, gaming, and motivation.

All proactive dialogues start with an intent that contains the External_ prefix. Daily activity and nutrition are dialogues started by the robot to ask about the user’s daily routines and nutrition habits. The responses of the users are stored in the DE to personalize future interactions with the system involving these routines. Some time-based proactive dialogues are set up at different times of the day to ask about the user’s feelings and to break up the loneliness of the user by starting dialogues about different topics. The initiation of the dialogues is conditioned by the presence of the user in the same room as the robot. The robot always starts by greeting and asking for permission to talk in the case of a proactive dialogue.

**Figure 10:**
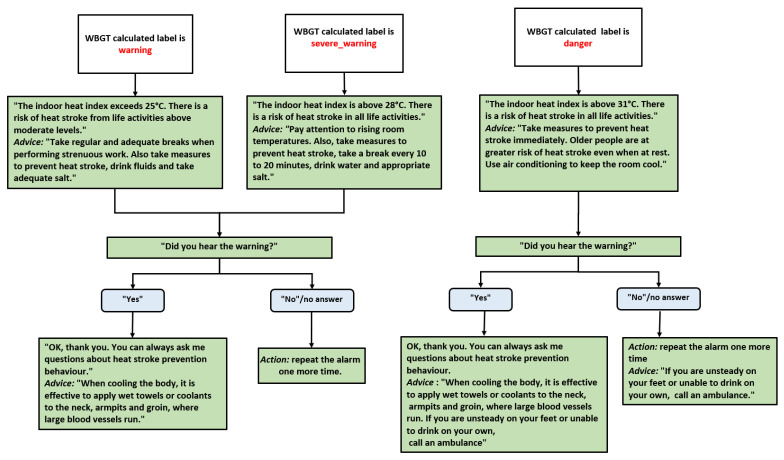
Dialogues based on the WBGT calculated label: warning, severe_warning, and danger. The green color indicates the dialogue of the virtual coach, while the gray color refers to the user’s answer.

The diagram in Figure 10 outlines a decision tree within the e-VITA virtual coaching system, strategically designed for heat stroke prevention dialogues using the WBGT index. This user-friendly decision tree provides specific virtual coach actions and advice for various WBGT ranges, enhancing user awareness and safety.

The decision tree systematically guides users through different scenarios based on the WBGT index, ensuring appropriate actions are taken to prevent heat-related risks. It incorporates a repetitive alarm as a reminder mechanism, reinforcing the importance of user engagement. The advice provided escalates in accordance with the severity of the temperature conditions, with a particular focus on the heightened vulnerability of older individuals to heat stroke.

The decision tree customizes its responses according to three distinct WBGT labels, i.e., warning (25 °C ≤ WBGT < 28 °C), severe warning (28 °C ≤ WBGT < 31 °C), and danger (WBGT ≥ 31 °C), ensuring that users receive information relevant to the specific severity of the environmental conditions. The inclusion of the question “Did you hear the warning?” followed by the appropriate actions ensures user engagement. The repetition of the alarm in the case of a negative response is implemented in situations where users might miss or not hear the initial warning. The advice provided for each WBGT range is proactive, offering preventive measures, such as taking breaks, staying hydrated, and adjusting activities. As the WBGT index increases, the advice becomes more reactive and urgent, with specific recommendations for dealing with higher temperatures, including the use of air conditioning and, in extreme cases, the suggestion to call an ambulance.

### 4.2. Adaptive Dialogues Connected to the EDS

**Figure 11:**
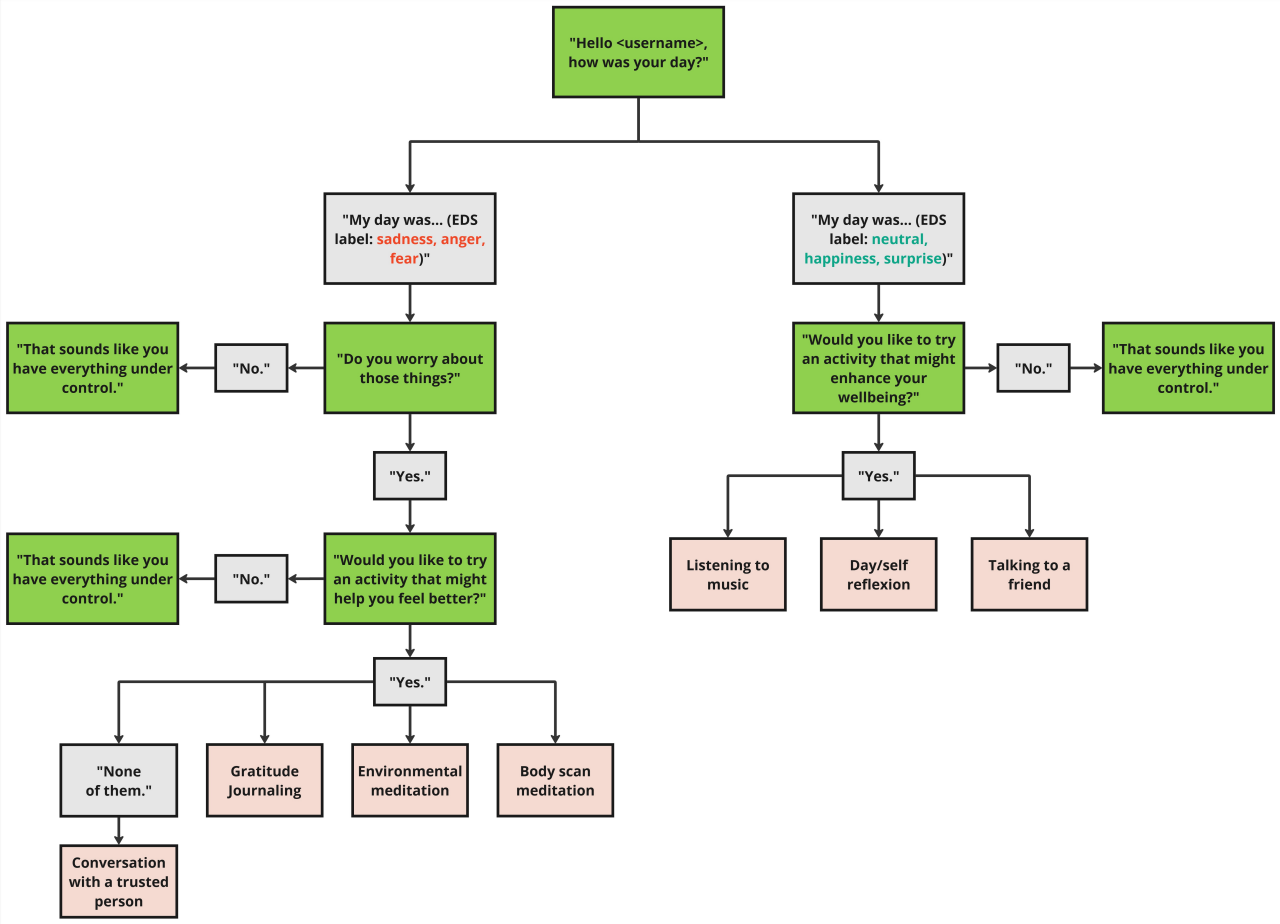
Course of the dialogue based on the emotions sadness, anger, fear, neutral, happiness and surprise. The green color represents the coach´s turns, while the gray color indicates the user´s turns. The salmon color refers to the selectable interventions.

As previously discussed, a proactive dialogue framework was designed that adapts to the emotional state of the user based on the outcomes of the Emotion Detection system (EDS). The course of the dialogue is guided by the labels provided by the EDS’s SER Model. The dialogue approach in “External_How_Was_Your_Day?” outlines a series of speech-based interactions between the user and the coaching system, facilitated by the analysis of the acoustic properties of the user’s speech and decisions. An outline of the decision tree within the e-VITA virtual coaching system course of the dialogue is presented in Figure 11.

The interaction begins with the system greeting the user (e.g., “Hello -username-, how was your day?”) to initiate a friendly and inviting conversation. This initial step elicits speech data for the subsequent acoustic speech analysis.

After the user shares details about their day, the coaching system analyses the response, focusing on acoustic cues to identify the user’s basic emotions. If emotions like happiness, surprise, or neutral are detected, the user is presented with the options of engaging in conversation with a friend, practicing self-reflection, or listening to music. Self-reflection has been shown to increase awareness of positive experiences [48], while listening to music is associated with improvements in overall well-being [49].

**Figure 12:**
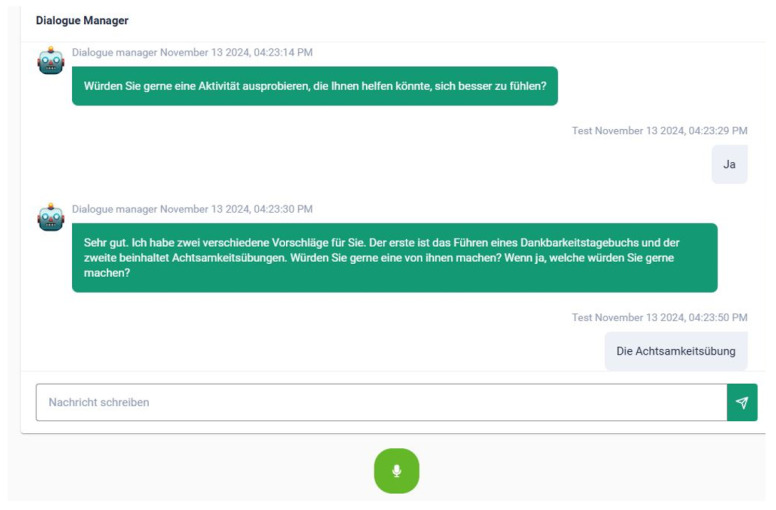
Example screenshot of the dialogue “How Was Your Day” in the e-VITA dashboard. This figure provides a possible exchange between the virtual coach and the user within the e-VITA dashboard. Translation: Coach: Would you like to try an activity that could help you feel better? User: Yes. Coach: Very well. I have two different suggestions for you. The first is to keep a Gratitude Journal and the second involves Mindfulness Exercises. Would you like to try one of these? If so, which one would you like to try? User: The Mindfulness Exercises.

If emotions such as anger, fear, or sadness are detected, the system offers targeted assistance. It first asks about the user’s concerns (e.g., “Do you worry about those things?”) to assess their openness to the proposed interventions. The user can then choose between Gratitude Journaling or Mindfulness Exercises if they like. Each intervention is designed to address or enhance emotional well-being. An exemplary screenshot of such a dialogue in the e-VITA dashboard can be seen in Figure 12.

In the case of choosing the Gratitude Journal, users are prompted to identify up to five things they are grateful for from the past day. Research indicates that gratitude journaling positively impacts well-being by enhancing emotional and social well-being [50], improving relationships, and promoting prosocial behavior [51]. Upon completing this exercise, the user has the option to set a reminder, including a timer, with the assistance of the coaching system to ensure the daily repetition of this beneficial practice.

In the case of choosing the mindfulness exercise, the user is subsequently presented with the option to engage in either a Body Scan Meditation or an Environmental Meditation. The Body Scan Meditation involves sequentially focusing on different parts of the body to increase body awareness and promote relaxation, helping individuals become more attuned to physical sensations and develop a sense of calm [52]. Environmental Meditation focuses on the surroundings during meditation, cultivating a sense of tranquillity and connectedness with the environment.

Mindfulness in general, as documented in existing research, enhances the capacity to respond to the present moment rather than react impulsively. Studies have shown that this practice reduces suffering and promotes well-being by fostering non-judgmental engagement with all experiences, whether positive, negative, or neutral [53].

If the user reports feeling unwell but declines to engage in an intervention, the coaching system recommends seeking a conversation with a trusted individual, such as a family member or friend. In the absence of available personal contacts, the system advises contacting a help hotline. This interaction enables users to share feelings, receive support, and gain new perspectives, thereby enhancing emotional support and overall well-being.

While only SER is employed in this specific dialog, the development of VER opens possibilities for future applications where combining SER and VER could deliver a more comprehensive emotional assessment. Scenarios where users are more stationary, or where smart home environments include cameras that capture facial expressions from a distance, could harness both verbal and facial cues to create a richer emotional profile and enhance personalization.

## 5. Conclusions and Future Work

In this paper, we explored the role of sensor technology in the e-VITA project, focusing on two important components of the e-VITA virtual coach: the data fusion and emotion recognition models. The data fusion model integrates heterogeneous data about the user’s activities and environment, while emotion recognition plays a crucial role in understanding the user’s mental state and tailoring the coach’s responses accordingly.

The Data Fusion Platform (DFP) collects data from a variety of smartphone and motion tracker sensors and calculates real time labels for each of the user’s activities. It also measures temperature, humidity, and CO2 and noise levels using an indoor climate monitoring system.

Our experiments show the need for heterogeneous data fusion due to the diverse nature of the data sources. We employed a flexible architecture framework involving machine learning-based HAR [6] to handle data heterogeneity issues. Issues with data fusion were explored especially in the area of user localization using a PIR sensor network and actimetry signals from wearable devices.

The DPF operation is illustrated through the following two key examples:

1. Environmental monitoring for heat stroke warnings;
2. A proactive notification system that alerts users to important events.

To initiate proactive dialogues based on predicted user situations, the e-VITA system employs an Event Processing/Rule-based approach. This is implemented as a notification system which is integrated into the communication scheme among the different components of the e-VITA system. The signals were integrated for interaction with Knowledge Graphs and the Dialogue Manager, using combined algorithmic and architectural principles.

The data fusion model for activity prediction assumes that there is one motion sensor in each room. However, in practice, this was a limitation for test scenarios, since some users did not allow the installation of several sensors in their home, so the tested scenarios were limited since the users were not equipped with a sufficient number of motion sensors.

The Emotion Detection System (EDS) includes voice-based emotion detection as well as visual emotion detection using state-of-the-art Convolutional Neural Network technology. The modules were implemented and evaluated separately, and the paper discusses these implementations and presents the results of the evaluations.

The speech emotion detection uses a deep neural network model for emotion classification, constructed from a two-dimensional time distributed convolutional neural network (2D-CNN) and a Long Short-Term Memory (LSTM) network. For visual emotion recognition, we developed and tested two frameworks as follows: The Conventional Framework and Shallow Convolutional Neural Networks.

A use case was developed specifically to evaluate the speech-based interaction, in which the coaching system asked the user about their day. We used the Speech Emotion Recognition model, implemented in the EDS, to assess the user’s emotional state based on their verbal responses.

In this article we have discussed the application of the data fusion and emotion recognition models in a coaching dialogue framework based on the specifications of the e-VITA project. Considering future development, some limitations of the work can also be mentioned. For instance, the data fusion model for activity prediction assumes that there is one motion sensor in each room. In practice, however, this was a limitation for test scenarios, since some users did not allow the installation of several sensors in their homes. Thus, the tested scenarios were limited since the users were not equipped with a sufficient number of motion sensors.

Regarding the CNN-based emotion detection model, this was trained on static images rather than videos, so it does not account for temporal dynamics or subtle changes in facial expressions over time. This could impact its performance in real-world scenarios where emotions evolve dynamically. Additionally, the model was trained and validated on seven emotions from the FER 2013 dataset, but it was evaluated on a subset of emotions, such as “Happy” and “Neutral”, which could limit its implications.

In future work, it will be important to integrate the two example use cases—environmental modeling and proactive notification system—more thoroughly with the dialogue capabilities of the e-VITA virtual coach. This includes modeling the use of environmental and user-related labels by the Dialogue Manager and the better modeling of the connections between human emotions and verbal communication. Moreover, it is important to pay attention to the limitations discussed above so as to be able to assess the implications of the models on the overall interaction.

The e-VITA virtual coach has been so far evaluated in a multinational proof-of-concept study across Europe and Japan, with results indicating the potential of AI assistants to promote active and healthy aging.

Another important area for future work is ethics and user privacy, while monitoring user activities is crucial for providing proactive assistance, it may also raise privacy concerns. A good balance can be achieved through transparency in the use of the data and by securing access to the data in such a way that it supports the user’s trust in the system. In the e-VITA virtual coach, the Digital Enabler ensured the safe and secure storage of the user’s private information, and these considerations should also be extended to dialogue modeling, which needs to take care of personalized information presentation as well as the use of this information in interactive situations where spoken dialogues may unintentionally reveal private information to other participants in the situation.

Overall, the insights gained from the e-VITA project regarding the use of sensor and emotion-based information to coach older adults towards an active and healthy lifestyle can be considered invaluable, as they provide a solid basis for future research and development in healthcare applications.

## Figures and Tables

**Table 1:** Evaluation performance of AU + SVM and the Shallow CNN on each annotated recording session individually. TN refers to the participant number. The mean accuracy of AU+SVM is 22% and the mean accuracy of the Shallow CNN is 49%.

Session Name	AU + SVM	Shallow CNN
TN1 Wizard	31.25 %	62.5%
TN2 Evita	11.11%	44.4%
TN2 Wizard	14.28%	42.85%
TN3 Evita	20.00%	20.00%
TN5 Evita	33.00%	75.00%
Mean ± std	21.92% ± 8.81	48.95% ± 18.75

**Table 2:** Performance evaluation of Shallow CNN on CISEVCSFN.

Weighted Average	Precision	Recall	F1-Score
	0.78	0.65	0.71

## Data Availability

Data are contained within the article.
